# Identification of stably expressed reference genes for expression studies in *Arabidopsis thaliana* using mass spectrometry-based label-free quantification

**DOI:** 10.3389/fpls.2022.1001920

**Published:** 2022-09-29

**Authors:** Sau-Shan Cheng, Yee-Shan Ku, Ming-Yan Cheung, Hon-Ming Lam

**Affiliations:** Centre for Soybean Research of the State Key Laboratory of Agrobiotechnology and School of Life Sciences, The Chinese University of Hong Kong, Shatin, Hong Kong SAR, China

**Keywords:** pathogen infection, jasmonic acid, salicylic acid, abscisic acid, expression study, label-free quantification, RT-qPCR, reference gene

## Abstract

*Arabidopsis thaliana* has been used regularly as a model plant in gene expression studies on transcriptional reprogramming upon pathogen infection, such as that by *Pseudomonas syringae* pv. *tomato* DC3000 (*Pst* DC3000), or when subjected to stress hormone treatments including jasmonic acid (JA), salicylic acid (SA), and abscisic acid (ABA). Reverse transcription-quantitative polymerase chain reaction (RT-qPCR) has been extensively employed to quantitate these gene expression changes. However, the accuracy of the quantitation is largely dependent on the stability of the expressions of reference genes used for normalization. Recently, RNA sequencing (RNA-seq) has been widely used to mine stably expressed genes for use as references in RT-qPCR. However, the amplification step in RNA-seq creates an intrinsic bias against those genes with relatively low expression levels, and therefore does not provide an accurate quantification of all expressed genes. In this study, we employed mass spectrometry-based label-free quantification (LFQ) in proteomic analyses to identify those proteins with abundances unaffected by *Pst* DC3000 infection. We verified, using RT-qPCR, that the levels of their corresponding mRNAs were also unaffected by *Pst* DC3000 infection. Compared to commonly used reference genes for expression studies in *A. thaliana* upon *Pst* DC3000 infection, the candidate reference genes reported in this study generally have a higher expression stability. In addition, using RT-qPCR, we verified that the mRNAs of the candidate reference genes were stably expressed upon stress hormone treatments including JA, SA, and ABA. Results indicated that the candidate genes identified here had stable expressions upon these stresses and are suitable to be used as reference genes for RT-qPCR. Among the 18 candidate reference genes reported in this study, many of them had greater expression stability than the commonly used reference genes, such as *ACT7*, in previous studies. Here, besides proposing more appropriate reference genes for Arabidopsis expression studies, we also demonstrated the capacity of mass spectrometry-based LFQ to quantify protein abundance and the possibility to extend protein expression studies to the transcript level.

## Introduction

Pathogen infection of plants has been a major cause of yield loss in agriculture (Gorshkov and Tsers, 2022). Upon pathogen infection, plants perceive the signal elicited by the secretions from the pathogens known as pathogen-associated molecular patterns (PAMPs) or microbe-associated molecular patterns (MAMPs; Medzhitov and Janeway, 1997), through pattern recognition receptors (PRRs). The signal perception triggers a series of defense responses including the generation of reactive oxygen species (ROS), fluctuations in the cellular calcium level, activation of proteins, such as mitogen-activated protein kinases (MAPKs), and GTP-binding proteins (G-proteins), and synthesis of stress hormones such as jasmonic acid (JA), salicylic acid (SA), and ethylene (Zhang and Zhou, 2010; Li et al., 2016b; Ku et al., 2020). These signaling events regulate the expressions of defense-related genes such as *Pathogenesis-Related* (*PR*) genes (Zhang and Zhou, 2010; Li et al., 2016b; Ku et al., 2020). PAMP-triggered immunity (PTI) describes the general pathogen resistance responses in plants (Zhang and Zhou, 2010; Li et al., 2016b; Ku et al., 2020). Some plants are known to have specific pathogen recognition mechanisms termed effector-triggered immunity (ETI; Dodds and Rathjen, 2010). ETI triggers hypersensitive response (HR) and usually leads to programmed cell death (PCD) at the area of infection to prevent the invading pathogens from spreading. In HR, ROS is produced to cause a series of cellular events including the disruption of cell membrane, thickening of the cell wall, and the production of stress hormones such as JA and SA. PTI and ETI lead to systemic acquired resistance (SAR) against a broad spectrum of pathogens (Dodds and Rathjen, 2010; Zhang and Zhou, 2010; Li et al., 2016b; Ku et al., 2020). The interactions among different signaling pathways, such as those of JA, SA, and abscisic acid (ABA), result in further complexity of the regulatory processes when under stress (Ku et al., 2018). These cellular events involve extensive transcriptional reprogramming (Li et al., 2016a). RNA sequencing (RNA-seq) has been used as the platform to study the global gene expression changes in plants upon pathogen infection (Zhu et al., 2013; Martin et al., 2016; Matic et al., 2016; Gupta and Senthil-Kumar, 2017; Poretti et al., 2021). Reverse transcription-quantitative polymerase chain reaction (RT-qPCR) is regarded as the gold standard for quantifying gene expressions due to its sensitivity, accuracy, and reproducibility (Gökmen-Polar, 2019). Although digital PCR (dPCR) has been shown to out-perform RT-qPCR in terms of sensitivity, accuracy, and reproducibility (Gökmen-Polar, 2019), RT-qPCR remains a more common approach for routine gene expression quantification due to the lower cost compared to dPCR. However, the accuracy of expression quantification by RT-qPCR is largely dependent on the expression stability of the reference gene used for normalization.

RNA-seq is commonly employed to mine reference genes for RT-qPCR (Yim et al., 2015; Kudo et al., 2016; Carmona et al., 2017; Zhou et al., 2017; Pombo et al., 2019). However, the accuracy of RNA-seq data has been a concern. PCR bias and GC content bias are hurdles for the accurate quantitative analysis of high-throughput sequencing data. In addition, the accuracy of quantitative gene expression analyses is highly influenced by the algorithms and pipelines for RNA-seq data analyses (Benjamini and Speed, 2012; Parekh et al., 2016; Corchete et al., 2020). In an attempt to assess the different RNA-seq data analysis pipelines, it was found that each of the 192 pipelines examined in the study had its advantages and disadvantages for quantitative gene expression analysis (Corchete et al., 2020).

Many previous researches have attempted to determine the correlation between mRNA and protein levels in plants. It was concluded that the correlation between mRNA levels and protein abundances is largely dependent on the plant species, tissue type, developmental stage, and stress condition of the plant (Nakaminami et al., 2014; Ponnala et al., 2014; Wang et al., 2017; Ding et al., 2020; Ren et al., 2022). Nevertheless, a significant correlation between mRNA and protein levels has been reported (Arefian et al., 2019; Ding et al., 2020; Shu et al., 2022; Zhu et al., 2022). Recent technologies have enabled high-throughput proteomic analyses through mass spectrometry, which does not involve amplification steps and thus may complement the limitations due to biased quantification in RNA-seq. In this study, we employed mass spectrometry-based label-free quantification (LFQ) to search for proteins that have stable abundances in *Arabidopsis thaliana* despite *Pseudomonas syringae* pv. *tomato* DC3000 (*Pst* DC3000) infection and tested the expression stability of their corresponding mRNAs by RT-qPCR. Compared to reference genes commonly used in previous studies, the mRNA levels of these proteins were generally more stable upon *Pst* DC3000 infection. We then extended the assessment of the stability of mRNA levels of these proteins to stress hormone treatments, including JA, SA and ABA. Altogether, 18 candidate reference genes were identified and tested. Using *A. thaliana* as the model, we revealed a set of more suitable reference genes for expression studies on *Pst* DC3000 infection and stress hormone treatments. We also demonstrated the advantage of using mass spectrometric analysis for mining genes which have stable protein and mRNA abundances upon various treatments.

## Materials and methods

### Plant materials and treatment conditions

For *Pst* DC3000 inoculation, *A. thaliana* plants (Col-0) were grown on Floragard potting soil in a growth chamber under these conditions: 22–24°C, light intensity 80–120 μE with a 16-h light:8-h dark cycle; relative humidity 70–80%. The rosette leaves of 5-week-old plants were inoculated with *Pst* DC3000 according to the protocol reported in previous studies (Cheung et al., 2013; Mi et al., 2013). At 0 day and 3 days post-inoculation (dpi), the aerial parts of the inoculated plants were collected, snap-frozen in liquid nitrogen, and stored at –80°C. The tissues of three individual plants were pooled as one biological replicate for total protein or total RNA extraction. A total of three biological replicates per treatment were collected for total protein extraction while two biological replicates per treatment were collected for total RNA extraction.

For JA treatment, the seeds of *A. thaliana* Col-0 were surface-sterilized by shaking in 100% household bleach for 3 min. After that, the bleach was removed, and the seeds were rinsed three times with sterilized water. The surface-sterilized seeds were then placed on Murashige & Skoog (MS) agar plates supplemented with 3% sucrose with or without 5 μM JA (Zhang et al., 2017). The seeded agar plates were then kept at 4°C in the dark for 2 days to break dormancy. After that, the plates were moved into a growth chamber under these conditions: 22°C–24°C, light intensity 80–120 μE with a 16-h light:8-h dark cycle, for 17 days. Then the seedlings were removed from the agar plates, frozen in liquid nitrogen, and stored at –80°C before total RNA extraction. Samples for two biological replicates per treatment were collected. Each biological replicate consisted of at least seven seedlings pooled together for total RNA extraction.

For SA treatment, the seeds of *A. thaliana* Col-0 were surface-sterilized as described above. The surface-sterilized seeds were then placed on MS agar plates without sucrose and with or without 50 μM SA (Sakurai et al., 2011; Sugano et al., 2016; Zhang et al., 2017). The seeded plates were then kept at 4°C in the dark for 2 days before being moved into a growth chamber under the same growth conditions as above for 17 days. After that, the seedlings were harvested from the agar plates, frozen in liquid nitrogen, and stored at –80°C before total RNA extraction. Two biological replicates per treatment were collected. Each biological replicate consisted of at least seven seedlings pooled together for total RNA extraction.

For ABA treatment, the seeds of *A. thaliana* Col-0 were sown on Floragard potting soil in a growth chamber under the same growth conditions as described above. Then the rosette leaves of 4-week-old plants were detached and floated on a perfusion solution (50 mM KCl, 10 mM MES, pH 7.0) under light for 2 h before being treated with ABA. ABA was first dissolved in 10% (v/v) methanol (MeOH) before being added to the perfusion solution to reach a final concentration of ABA at 10 μM and MeOH at 0.1% (v/v). The detached leaves were treated with ABA under light for 2 h. Then they were frozen in liquid nitrogen and stored at –80°C before total RNA extraction. Two biological replicates were sampled for each treatment, and each biological replicate consisted of the rosette leaves of at least three plants pooled together for total RNA extraction.

### Protein extraction and protein profile analysis by liquid chromatography–tandem mass spectrometry (LC–MS/MS)

Total protein was extracted from the plant samples according to a previously described protocol (Marx et al., 2016; Cheng et al., 2022), with minor modifications. The plant samples were ground to a fine powder in liquid nitrogen using mortar and pestle. Around 100 mg of the powder was resuspended in five volumes of total protein extraction buffer [290 mM sucrose, 250 mM Tris (pH 7.6), 50 mM Na pyrophosphate, 25 mM EDTA (pH 8.0), 25 mM NaF, 10 mM KCl, 1 mM (NH_4_)_6_Mo_7_O_24_, 1 mM phenylmethylsulfonyl fluoride (PMSF), and 1X Halt™ Protease Inhibitor Cocktail (Cat#78430, Thermo Fisher Scientific, Waltham, MA, United States)]. The plant proteins in the extract were precipitated using the chloroform/methanol method (Wessel and Flügge, 1984). After that, the protein pellet was lyzed with five volumes of lysis buffer (w/v) [8 M urea, 50 mM Tris–HCl (pH 8.0), 30 mM NaCl, 20 mM sodium butyrate, 1 mM CaCl_2_, and 1X Halt™ Protease Inhibitor Cocktail (Cat#78430, Thermo Fisher Scientific, Waltham, MA, United States)]. The protein concentration was determined using the Pierce™ BCA Protein Assay Kit (Cat#23225, Thermo Fisher Scientific, Waltham, MA, United States). Then, 10 μg of each protein sample was reduced with 5 mM dithiothreitol (DTT) at 37°C for 30 min, alkylated with 20 mM iodoacetamide at room temperature for 30 min, and underwent a final reduction with 5 mM DTT at 37°C for 30 min. Then the protein mixture was incubated with trypsin in a ratio of 1/20 (w/w) of the protein amount at 37°C overnight.

The peptides resulting from trypsin digestion were desalted using Pierce™ C18 Spin Column (Cat#89873, Thermo Fisher Scientific, Waltham, MA, United States), and each sample was analyzed independently for protein identification using LC–MS/MS. Five hundred nanograms of desalted peptides from each sample were injected into the LC Ultimate 3000 RSLCnano system equipped with a C-18 μ-precolumn (300-μm i.d. × 5 mm) with an Acclaim Pepmap RSLC nanoViper C-18 column (75 μm × 25 cm) coupled to the Orbitrap Fusion Lumos Tribrid mass spectrometer (Thermo Fisher Scientific, Waltham, MA, United States). The peptide samples were separated against the gradient profile with a 50°C chamber at a flow rate of 0.3 μLmin^−1^, using a mixture of ultrapure water with 1.9% acetonitrile and 0.1% formic acid as mobile phase A and ultrapure water with 2% acetonitrile and 0.1% formic acid as mobile phase B, with the following gradient profile setting in the LC: 0% mobile phase B for the initial 5 min; at 5–8 min, 0–6% mobile phase B; 8–48 min, 6–18% mobile phase B; 48–58 min, 18–30% mobile phase B; 58–65 min, 30–80% mobile phase B; and then at 65–75 min, 0% mobile phase B for re-equilibration of the column. Each desalted peptide sample was analyzed twice (as technical replicates) to eliminate the instrumental variations.

The raw data files were generated using Xcalibur software (Thermo Fisher Scientific, Waltham, MA, United States) for MS/MS identification using Proteome Discoverer v2.4 (Thermo Fisher Scientific, Waltham, MA, United States) against the *A. thaliana* protein database (TAIR10) with the built-in SEQUEST HT program at the following settings: MS precursor mass tolerance of 10 ppm, fragment mass tolerance of 0.02 Da, a maximum of 2 missed trypsin cleavage, fixed N-terminal protein acetylation (+42.011 Da), dynamic cysteine carbamidomethylation (+57.021 Da) and methionine oxidation (+15.995 Da). Peptide validation using the built-in Percolator program was accepted at a false discovery rate (FDR) with a *q*-value <0.01. Samples were compared using the LFQ method according to the protocol in Proteome Discoverer v2.4 (Thermo Fisher Scientific, Waltham, MA, United States). The proteomic dataset was deposited to PRIDE (PRoteomics IDEntifications Database; Project accession: PXD035677). The grouping of the protein samples from different treatments was presented using PCA plot (Supplementary Figure S1). Proteins appearing in all biological replicates with an adjusted value of *p* > 0.05 for the difference in the abundance (using the Benjamini-Hochberg correction) between 0 and 3 dpi with *Pst* DC3000, i.e., no significant difference in abundance, were short-listed as possible reference gene candidates. Among the short-listed candidates, 18 proteins were highly ranked as stably expressed by Proteome Discoverer v2.4 (Thermo Fisher Scientific, Waltham, MA, United States) and were included in the final list of candidate reference genes (Supplementary Table S1).

### Primer design

Primers for RT-qPCR were designed according to the mRNA sequences of the candidate reference genes. The primer specificity was determined by the Primer-BLAST function on the NCBI platform against the *A. thaliana* genome (taxid: 3702) and validated by melting curve analysis after RT-qPCR (Supplementary Figure S2). For references genes commonly used in previous studies, the primer sequences were adopted from the corresponding publications (Czechowski et al., 2005; Jeong et al., 2011; Huang et al., 2013; Chen et al., 2014; Cheong et al., 2014; Cuéllar Pérez et al., 2014; Kim and Hwang, 2014; Zhang et al., 2015; Huot et al., 2017; Jia et al., 2018; Wu et al., 2019; Cui et al., 2021; Romero-Pérez et al., 2021; Gao et al., 2022). The primer sequences are listed in Supplementary Table S1.

### Total RNA extraction, cDNA synthesis, and RT-qPCR

The plant samples were ground in liquid nitrogen to a fine powder. After that, total RNA was extracted using Trizol™ Reagent (Cat#15596018, Thermo Fisher Scientific, Waltham, MA, United States) according to the manufacturer’s protocol. Then, the RNA was quantitated using the Qubit™ RNA Broad Range (BR) Assay Kit (Cat#Q10211, Thermo Fisher Scientific, Waltham, MA, United States) with the use of Qubit 2.0 Fluorometer (Cat#Q32866, Thermo Fisher Scientific, Waltham, MA, United States) according to the manufacturer’s protocol. For *Pst* DC3000 infection and ABA treatment, 320 ng of total RNA was used for cDNA synthesis. For JA and SA treatments, 640 ng of total RNA was used for cDNA synthesis. For cDNA synthesis, the RNA was first treated with DNase I according to the manufacturer’s protocol (Cat#18068015, Thermo Fisher Scientific, Waltham, MA, United States). The DNase I-treated RNA was then subjected to cDNA synthesis using the High-Capacity cDNA Reverse Transcription Kit with RNase Inhibitor (Cat#4374966, Thermo Fisher Scientific, Waltham, MA, United States) according to the manufacturer’s protocol, with the random primers being replaced by oligo(dT)_20_ to make up a final concentration of 20 μM oligo(dT)_20_. After that, the cDNA was diluted 30 folds before being used for qPCR. For qPCR, 3 μl diluted cDNA was added to a 20-μL qPCR reaction mix with 1X SsoAdvanced Universal SYBR Green Supermix (Cat#1725270, Bio-Rad, Hercules, CA, United States) and 0.15 μM each of forward and reverse primers. Quantitative PCR and melting curve analyses (from 95°C to 65°C) were performed using a CFX96 Touch Real-Time PCR system (Bio-Rad, Hercules, CA, United States).

### Stability analysis of the candidate reference genes and reference genes commonly used in previous studies

In each treatment, three technical replicates of the qPCR were performed for each primer pair. The six *C_t_* values from the six technical replicates in total of two biological replicates were used as the inputs for stability analyses using programs including geNorm *via* the R-based package ctrlGene (ver. 1.0.0; Vandesompele et al., 2002; Zhong, 2019), Normfinder *via* RefFinder (Andersen et al., 2004; Xie et al., 2012), BestKeeper (Pfaffl et al., 2004), the comparative Δ*C_t_* method (Silver et al., 2006), and RefFinder (Xie et al., 2012) with default parameters.

## Results

### Identification of stably expressed proteins unaffected by *Pst* DC3000 infection

**Figure 1:**
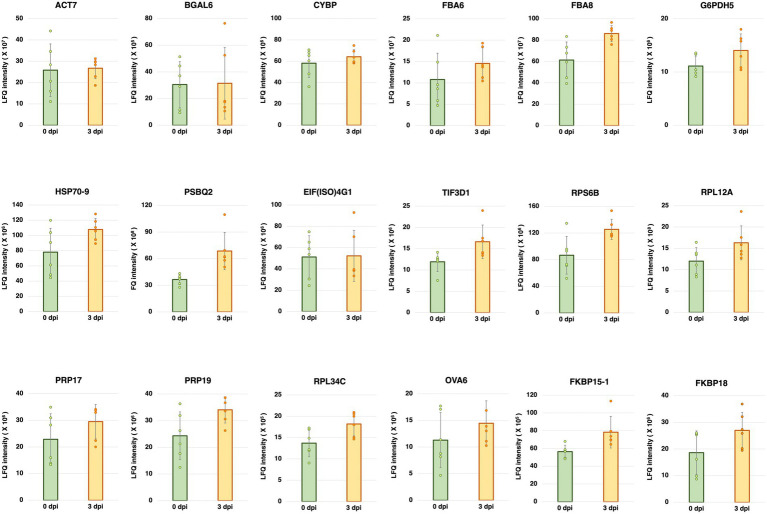
Label-free quantification (LFQ) intensities of the candidate reference genes having stable abundances between 0 and 3 days post-inoculation (dpi) with *Pseudomonas syringae* pv. *tomato* DC3000 (*Pst* DC3000) in 5-week-old *A. thaliana* ecotype Col-0. The protein abundance levels of these candidate reference genes, calculated from LFQ using Proteome Discoverer v2.4 (Thermo Fisher Scientific, Waltham, MA, United States), were not statistically different between 0 and 3 dpi (*p* > 0.05). Three individual plants were pooled as one biological replicate, each with two technical repeats of LFQ analysis. Error bar represents the standard deviation of a total of six technical repeats based on three biological replicates.

Five-week-old *A. thaliana* plants (Col-0) were inoculated with *Pst* DC3000. Total protein was extracted from the inoculated plants at 0 and 3 dpi, and then subjected to mass spectrometry-based LFQ analyses. Eighteen proteins were found to have stable abundances between 0 and 3 dpi (Figure 1). The gene names and accession numbers corresponding to the proteins are listed in Supplementary Table S1. The abundance of the proteins corresponding to 12 reference genes commonly used in previous studies (Czechowski et al., 2005; Jeong et al., 2011; Huang et al., 2013; Chen et al., 2014; Cheong et al., 2014; Cuéllar Pérez et al., 2014; Kim and Hwang, 2014; Zhang et al., 2015; Huot et al., 2017; Jia et al., 2018; Wu et al., 2019; Cui et al., 2021; Romero-Pérez et al., 2021; Gao et al., 2022) were also investigated using the protein dataset. The gene names and accession numbers are listed in Supplementary Table S1. Among the 12 reference genes commonly used in previous studies, ACT1, ACT8, PP2AA3, UBQ5, UBC9, and TIP41 were found from the protein dataset. The abundances of ACT1, ACT8, PP2AA3, and UBQ5 did not show statistically significant difference between 0 dpi and 3 dpi, the abundance of UBC9 was higher at 0 dpi compared to 3 dpi, while the abundance of TIP41 was too low to be quantified (Supplementary Figure S3). The protein abundance could be found from the protein dataset deposited to PRIDE (PRoteomics IDEntifications Database; Project accession: PXD035677). The chromatograms of the LC/MS–MS analyses are shown in Supplementary Figures S4, S5.

### Amplification specificity and efficiency

To test the abundance of the mRNAs corresponding to the stably expressed proteins shown in Figure 1, specific primers were designed based on their mRNA sequences. The specificity of each primer pair was first determined using the Primer-BLAST function on the NCBI platform against the *A. thaliana* genome (taxid: 3702) and validated by melting curve analyses after RT-qPCR (Supplementary Figure S2).

**Figure 2:**
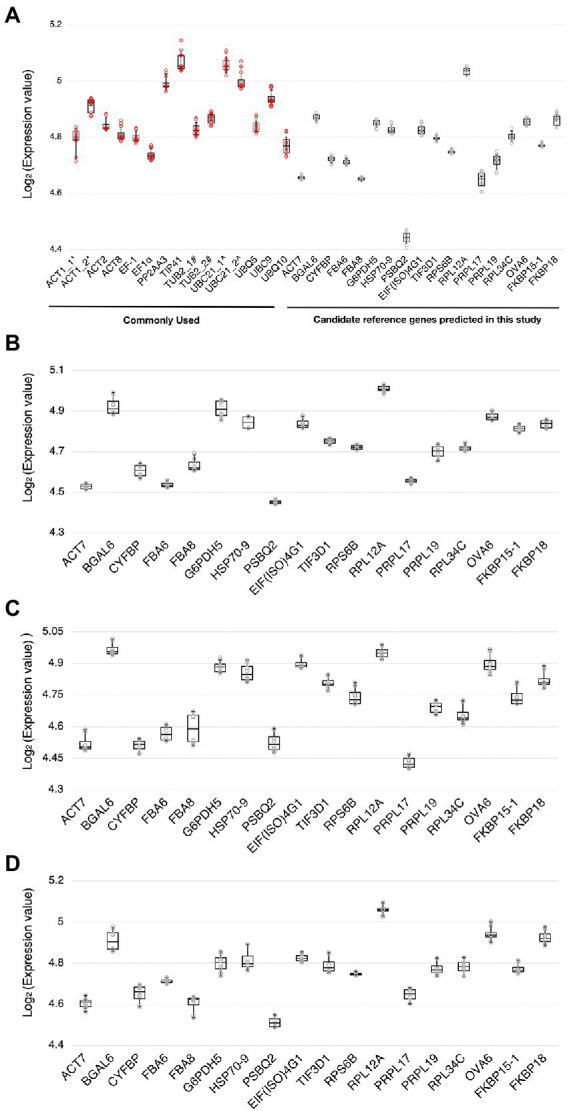
Ranges of expression levels of the candidate reference genes and reference genes commonly used in previous studies. **(A)** A boxplot showing the range of expression values [log_2_(*C_t_* value)] of the candidate reference genes and the reference genes commonly used in previous studies obtained by RT-qPCR when subjected to *Pst* DC3000 at 0 dpi and 3 dpi. **(B–D)** Boxplots showing the range of expression values [log_2_(*C_t_* value)] of the candidate reference genes when subjected to jasmonic acid (JA) **(B),** salicylic acid (SA) **(C)**, and abscisic acid (ABA) **(D)** treatments and the corresponding mock control treatments. The solid line inside each box represents the median expression value and the lower and upper edges of the boxes denote the 25^th^ and 75^th^ percentiles, while the whiskers represent the maximum and the minimum values. Each dot represents the expression value calculated from each technical replicate of RT-qPCR. Three technical repeats were performed for each biological replicate, with two biological replicates in total.

Using RT-qPCR, we analyzed the expression levels of the 18 candidate reference genes and 12 reference genes commonly used in previous studies (Supplementary Table S1; Czechowski et al., 2005; Jeong et al., 2011; Huang et al., 2013; Chen et al., 2014; Cheong et al., 2014; Cuéllar Pérez et al., 2014; Kim and Hwang, 2014; Zhang et al., 2015; Huot et al., 2017; Jia et al., 2018; Wu et al., 2019; Cui et al., 2021; Romero-Pérez et al., 2021; Gao et al., 2022) at 0 and 3 dpi with *Pst* DC3000 inoculation. We also evaluated the expression stability of these candidate reference genes in stress hormone treatments, including JA, SA, and ABA. The distributions of the *C*_*t*_ values of the candidate reference genes were shown by boxplots (Figure 2). In *Pst* DC300 infection, the average expression values of the reference genes commonly used in previous studies ranged from the log_2_ values of 4.71 and 5.14 (Figure 2A), which are equivalent to *C*_*t*_ values of 26.26 to 35.38. Meanwhile, the average expression values of the candidate reference genes predicted in this study ranged from the log_2_ values of 4.41–5.05 (Figure 2A), which are equivalent to *C*_*t*_ values of 21.20-33.21. In *Pst* DC3000 infection, the expression levels of the reference genes commonly used in previous studies are generally lower than those of the candidate reference genes predicted in this study. Considering all the treatments, the average expression values of the candidate reference genes predicted in this study were in the range of log_2_ values of 4.40–5.10, equivalent to the *C_t_* values of 21.12–34.22. Based on the *C_t_* values obtained from RT-qPCR and the LFQ intensities obtained from the mass spectrometry-based proteomic analysis, the mRNA level was found to be positively correlated to the protein abundance (Supplementary Figure S6).

### Stability analysis of the mRNAs

#### Expression stability comparison between the candidate reference genes and reference genes commonly used in previous studies upon *Pst* DC3000 infection

To compare the expression stabilities of the 18 candidate reference genes predicted in this study and those of the 12 reference genes commonly used in previous studies, the expression stabilities were ranked using geNorm (Vandesompele et al., 2002) and RefFinder (Xie et al., 2012).

##### Stability analysis by geNorm

**Figure 3:**
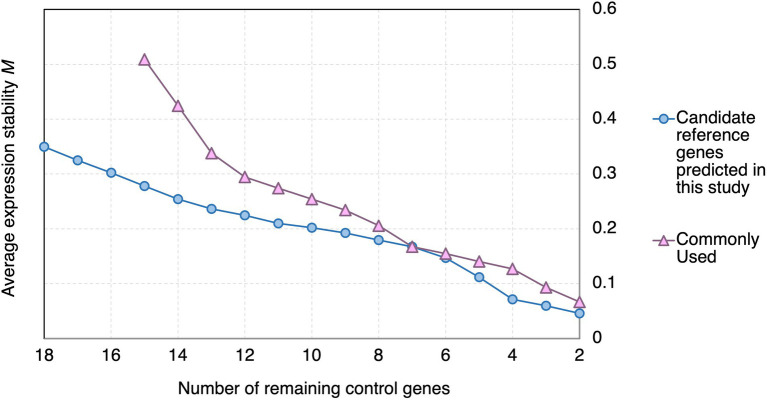
geNorm analysis of the average stability values of the candidate reference genes predicted in this study and reference genes commonly used in previous studies upon *Pst* DC3000 infection. Average expression stability values (*M*) of the remaining control genes during the step exclusion of the least stable control among the reference gene candidate upon *Pst* DC3000 infection using geNorm. The higher the average *M*, the lower rank of the expression stability of the reference gene candidate.

In geNorm analysis, the expression stability of the candidate reference genes was calculated using pairwise comparisons (Vandesompele et al., 2002), and presented in the form of the average expression stability value (*M*), with a lower *M* representing a higher stability (Vandesompele et al., 2002). Genes with *M* < 1.5 are commonly considered stably expressed (Vandesompele et al., 2002; Walker et al., 2009; Jin et al., 2019; Fu et al., 2022). All the candidate reference genes and the reference genes commonly used in previous studies showed *M* < 1.5 (Figure 3). However, the *M* values of the candidate reference genes reported in this study were generally lower than those of the reference genes commonly used in previous studies (Figure 3).

**Figure 4:**
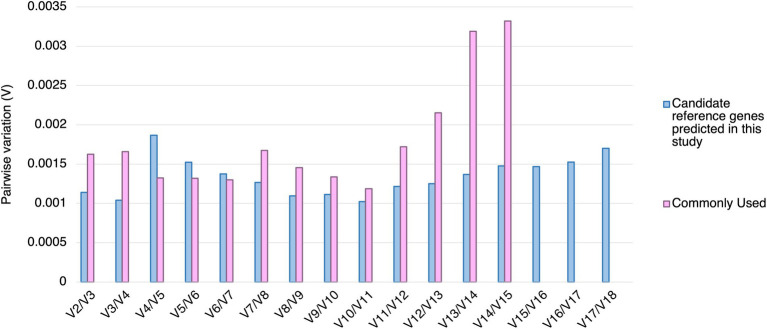
Pairwise variation (V) of the candidate reference genes predicted in this study and reference genes commonly used in previous studies calculated by geNorm. The pairwise variation representing (V_n_/V_n + 1_). The threshold value of V for accessing the optimal number of the reference genes for RT-qPCR normalization is 0.15. n represents the number of reference gene required for expression normalization in RT-qPCR.

Using geNorm analysis, the optimal number of reference genes required for expression normalization in RT-qPCR was determined by the pairwise variation calculation (V_n_/V_n + 1_), in which n represents the number of reference genes required for expression normalization in RT-qPCR. All the candidate reference genes and the reference genes commonly used in previous studies showed V_n_/V_n + 1_ values smaller than 0.15, which is the cut-off value indicating the expression stability (Vandesompele et al., 2002; Figure 4). However, the candidate reference genes predicted in this study showed general smaller V values compared to the reference genes commonly used in previous studies (Figure 4).

##### Comprehensive stability analysis by RefFinder

**Figure 5:**
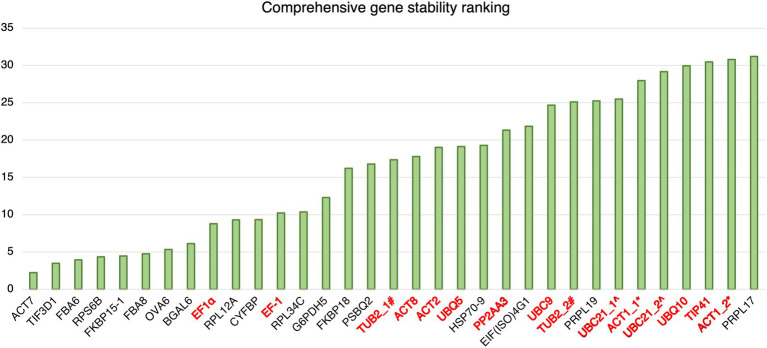
Comprehensive gene stability ranking of the predicted reference gene candidates and the commonly used reference gene for *Pst* DC3000 inoculation. The stability ranking was generated using the RefFinder. A lower stability ranking refers to a higher stability of the reference gene. Gene names in black: candidate reference genes reported in this study; gene names in red: reference genes commonly used in previous studies. ^#^TUB2_1 and TUB2_2 refer to two different primer pairs for TUB2 (Wu et al., 2019; Gao et al., 2022). ^^^UBC21_1 and UBC21_2 refer to two different primer pairs for UBC21 (Cuéllar Pérez et al., 2014; Gao et al., 2022)^. *^ACT1_1 and ACT1_2 refer to two different primer pairs for ACT1 (Kim and Hwang, 2014; Zhang et al., 2015).

The results of geNorm analysis suggest that the candidate reference genes predicted in this study are generally more stable than the reference genes commonly used in previous studies upon *Pst* DC3000 infection. The stabilities were further analyzed using RefFinder (Xie et al., 2012), which calculates the comprehensive gene stability by integrating the algorithms of geNorm, NormFinder, BestKeeper, and the comparative Δ*C_t_* method (Xie et al., 2012). The result from RefFinder also suggests that the candidate reference genes reported in this study are generally more stable than the reference genes commonly used in previous studies upon *Pst* DC3000 infection (Figure 5). Among all the genes, *ACT7*, *TIF3D1*, and *RPS6B*, which are candidate reference genes reported in this study, were the most stably expressed upon *Pst* DC3000 infection (Figure 5).

#### Expression stability analyses of the candidate reference genes in various treatments

The above results suggest that the expressions of the candidate reference genes predicted in this study are generally more stable than those of the reference genes commonly used in previous studies. In addition to *Pst* DC3000 infection, we further analyzed the expression stabilities of the candidate reference genes in stress hormone treatments including JA, SA, and ABA. To rank the expression stability of the 18 candidate references genes under different treatments, programs including geNorm (Vandesompele et al., 2002), NormFinder (Andersen et al., 2004), BestKeeper (Pfaffl et al., 2004), and RefFinder (Xie et al., 2012) were used.

##### Stability analysis by geNorm

**Figure 6:**
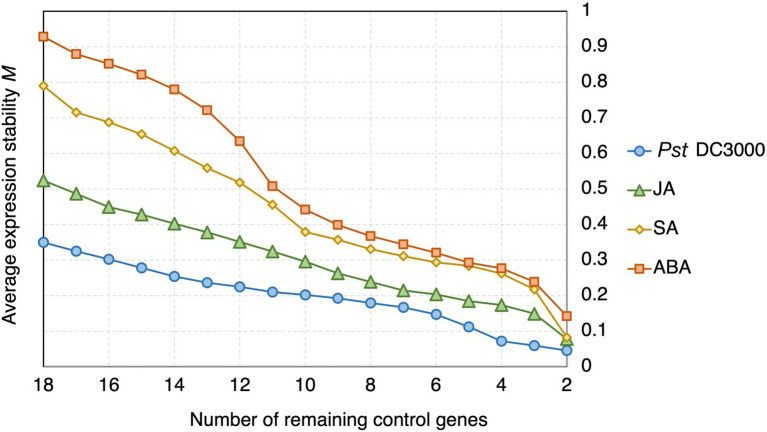
geNorm analysis of the average expression stability values of the candidate reference genes under *Pst* DC3000, JA, SA, and ABA treatments. Average expression stability values (*M*) of the remaining control genes were calculated by a stepwise exclusion of the least stable control gene among the reference gene candidates under each treatment using geNorm. The higher the *M* value, the lower is the ranking of the reference gene candidate in terms of expression stability.

**Figure 7:**
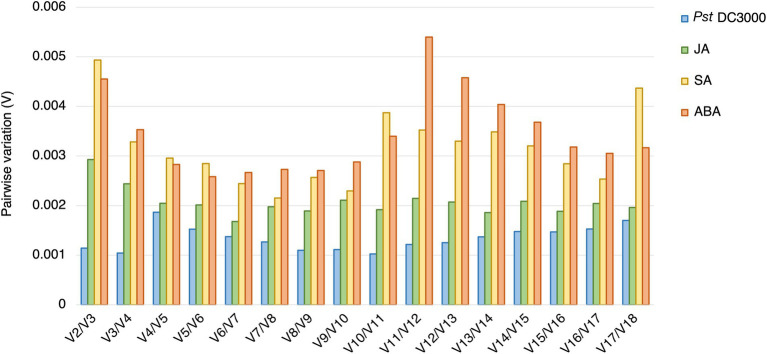
Pairwise variations (V) of the candidate reference genes. The pairwise variations (V) of the candidate reference genes calculated using geNorm, and V = V_n_/V_n + 1_, where *n* is the number of reference genes required for expression normalization in RT-qPCR. The threshold value of V for achieving the optimal number of reference genes for RT-qPCR normalization is 0.15. All the candidate genes had V values well below the threshold value under all the treatments, indicating a minimal number of reference genes from among these candidates is required for normalization in RT-qPCR.

In geNorm analysis, all the 18 candidate reference genes had *M* values <1 in all the different treatments (Figure 6; Supplementary Table S2). The *M* values in *Pst* DC3000 infection are generally lower than those in other treatment (Supplementary Table S2). In *Pst* DC3000 infection, among the candidate genes, *RPS6B* and *FKBP15-1* were the most stably expressed (*M* = 0.046) while *PRPL17* was the least stably expressed (*M* = 0.35; Supplementary Table S2). For gene expression analyses using RT-qPCR, multiple reference genes are usually required for accurate expression normalization if the expression stability of the reference gene is low (Vandesompele et al., 2002). Among the 18 candidate reference genes, all the V_n_/V_n + 1_ values were much lower than the cut-off value of 0.15 (Figure 7). Such results suggest that using two reference genes would be good enough, eliminating the need of a third one, for expression normalization (Vandesompele et al., 2002).

##### Stability analysis by NormFinder

The expression stability values for each candidate reference gene was also analyzed using the linear mixed-effect model-based NormFinder, in which the variations between the input *C_t_* values are considered in testing the gene expression stability (Andersen et al., 2004). Similar to geNorm, a higher stability value represents a lower expression stability of the reference gene candidate. The range of the stability values varied among treatments. It ranged between 0.041 and 0.528 under *Pst* DC3000 infection, between 0.088 and 0.765 with JA treatment, between 0.266 and 1.333 with SA treatment, and between 0.316 and 1.18 with ABA treatment (Supplementary Table S2). These results mean that the expression stability of the candidate reference genes varied under different treatments, but all had the highest stability upon *Pst* DC3000 infection.

##### Stability analysis by BestKeeper

The stability of the reference gene candidates was also tested using BestKeeper, in which the standard descriptive statistics for the genes are considered (Pfaffl et al., 2004). The BestKeeper algorithm suggests excluding those candidate reference genes with a standard deviation (SD) > 1.0 in *C_t_* due to low expression stability (Pfaffl et al., 2004; Piehler et al., 2010). Among all the candidate reference genes for all the treatments, only *FBA8* under SA treatment had an *SD* > 1 (*SD* = 1.125) in its *C_t_* values while all the other genes had their *SD* < 1 for all the treatments (Supplementary Table S2). Similar to the results from NormFinder, results from BestKeeper suggest that the expression stability of the reference genes varies under different treatments, but with the highest stability upon *Pst* DC3000 infection in general. Nevertheless, the results strongly suggest the stability of all the candidate genes was acceptable as reference genes under all the treatment conditions tested.

##### Stability analysis by the comparative Δ*C_t_* method

We also used the comparative Δ*C_t_* method (Silver et al., 2006) to evaluate the relative stability of the candidate reference genes. The method compares the relative expression fluctuation of the two reference gene by measuring differences between their Δ*C*_*t*_ values (Silver et al., 2006; Nagy et al., 2017). The average SD of the 18 candidate reference genes ranged from 0.26 to 0.55 for *Pst* DC3000 treatment, 0.39 to 0.82 for JA treatment, 0.62 to 1.39 for SA treatment and 0.73 to 1.33 for ABA treatment (Supplementary Table S2). Among all the candidates, *TIF3D1* was the most stably expressed in both *Pst* DC3000 and SA treatments while *RPS6B* and *FKBP15-1* were the most stable with JA and ABA treatments, respectively (Supplementary Table S2). Similar to the results from NormFinder and BestKeeper, the results in the comparative Δ*C*_*t*_ method showed that the stability of the reference genes varied with treatments, but they all had the highest expression stability upon *Pst* DC3000 infection.

##### Stability analysis by RefFinder

To gain a comprehensive view of the stability ranking of the 18 candidate reference genes, the results from geNorm, NormFinder, BestKeeper, and the comparative Δ*C_t_* method were integrated using RefFinder (Xie et al., 2012; Supplementary Table S2). The results showed that the candidate reference genes had different degrees of expression stability under different treatments (Supplementary Table S2). Based on the comprehensive ranking, *TIF3D1*, *RPS6B*, and *FKBP15-1* were the most stably expressed under *Pst* DC3000 treatment, *RPS6B*, *PSBQ2*, and *FKBP15-1* most stably expressed with JA treatment, *TIF3D1*, *CYFBP*, and *FBA6* most stably expressed under SA treatment, and *PRPL17*, *PSBQ2,* and *FKBP15-1* most stably expressed with ABA treatment (Supplementary Table S2).

To see whether a single reference gene would be suitable for expression normalization for samples treated with different stresses, we estimated the geometric means of the rankings from RefFinder of each reference gene under different treatment combinations and suggested the most suitable candidate reference genes under these treatments (Table 1).

**Table 1:** Appropriate *A. thaliana* reference genes for expression normalization under different combinations of treatments, including *Pseudomonaas syringae* pv. *tomato* DC3000 (*Pst* DC3000), jasmonic acid (JA), salicylic acid (SA), and abscisic acid (ABA).

Treatment(s)				Suggested reference genes		
*Pst* DC3000	JA	SA	ABA	*RPS6B* (1.41)	*TIF3D1* (2.71)	*PRPL19* (3.16)
*Pst* DC3000	JA	SA		*PRPL19* (2.55)	*RPS6B* (2.59)	*FKBP18* (2.99)
*Pst* DC3000	JA		ABA	*RPS6B* (1.57)	*PSBQ2* (2.78)	*RPL12A* (3.44)
*Pst* DC3000		SA	ABA	*FKBP15-1* (2.06)	*RPS6B* (2.3)	*PRPL19* (3.83)
*Pst* DC3000	JA			*RPS6B* (1.19)	*FKBP18* (2.3)	*PSBQ2* (3.31)
*Pst* DC3000		SA		*TIF3D1* (2.21)	*PRPL19* (2.38)	*G6PDH5* (3.6)
*Pst* DC3000			ABA	*RPS6B* (1)	*FBA6* (1.68)	*FKBP15-1* (3)
*Pst* DC3000				*RPS6B* (1.32)	*FKBP15-1* (2)	*FBA8* (2.45)
	JA	SA	ABA	*RPS6B* (2)	*ACT7* (2.24)	*PSBQ2* (2.66)
	JA	SA		*PRPL19* (2)	*ACT7* (2.28)	*RPS6B* (2.94)
	JA		ABA	*RPS6B* (1.63)	*PSBQ2* (2.11)	*ACT7* (3)
	JA			*RPS6B* (2.55)	*PSBQ2* (3.16)	*ACT7* (3.46)
		SA	ABA	*ACT7* (2.43)	*FKBP15-1* (3.46)	*RPS6B* (3.56)
		SA		*CYFBP* (2.17)	*TIF3D1* (2.34)	*EIF(ISO)4G1* (4.86)
			ABA	*FBA6* (2.06)	*RPS6B* (2.38)	*FKBP15-1* (3.08)

The geometric means of the RefFinder rankings of each candidate reference gene under the corresponding pair of treatment conditions are in brackets. The shade means blank.

## Discussion

In this study, we employed mass spectrometry-based LFQ and identified the *A. thaliana* proteins having stable abundances upon *Pst* DC3000 treatment. The candidate proteins fell into these categories: structural protein [ACT7 (Paez-Garcia et al., 2018)], basal metabolism-related proteins [BGAL6 (Dwevedi and Kayastha, 2010), CYFBP (Daie, 1993), FBA6 (Carrera et al., 2021), FBA8 (Lu et al., 2012), G6PDH5 (Sharkey and Weise, 2016), and PSBQ2 (Yi et al., 2006), protein-folding regulators HSP70-9 (Sung et al., 2001), FKBP15-1 and FKBP18 (Harrar et al., 2001)], and translation regulatory proteins [EIF(ISO)4G1 (Martínez-Silva et al., 2012), TIF3D1 (Raabe et al., 2019), RPS6B (Horiguchi et al., 2012), RPL12A, RPL17, RPL19, and RPL34C (Martinez-Seidel et al., 2020), and OVA6 (Berg et al., 2005)]. The results are consistent with the notion that genes involved in the maintenance of basal cellular functions tend to have stable expressions (Eisenberg and Levanon, 2013). It was therefore reasonable to expect the levels of the mRNAs encoding these proteins to also be relatively stable and that the study of expression stability upon pathogen infection could be applied to other treatments. The protein abundance and the mRNA level were found to be positively correlated (Supplementary Figure S6). In addition to the positive correlation, the slope of the line of best fit (Supplementary Figure S6) suggests that the mass spectrometry-based LFQ in proteomic analysis is more sensitive than RT-qPCR for expression quantitation. Such a high sensitivity of the mass spectrometry-based LFQ in proteomic analysis will enable accurate quantitation particularly if the experimental data fit the line of best fit well.

For the reference genes commonly used in previous studies, upon *Pst* DC3000 infection, the expression levels were generally lower than those of the candidate reference genes used in this study (Figure 2). The result is consistent with the lower abundance of the proteins compared to the proteins corresponding to the candidate reference genes reported in this study (Figure 1; Supplementary Figure S3). It is possible that proteins having higher abundances are more easily detectable by LC–MS/MS. In other words, the mass spectrometry-based proteomic analysis favors the detection of highly expressed proteins, which may imply the high levels of the corresponding mRNAs. The use of highly expressed genes for expression normalization in RT-qPCR facilitates the expression analysis when the input amount of cDNA or RNA is low.

The expression levels of the candidate reference genes were in a reasonably detectable range (Figure 2). The expression stability of the candidate reference genes was estimated using multiple programs including geNorm *via* the R-based package ctrlGene (ver. 1.0.0; Vandesompele et al., 2002; Zhong, 2019), Normfinder *via* RefFinder (Andersen et al., 2004; Xie et al., 2012), BestKeeper (Pfaffl et al., 2004) and the comparative Δ*C_t_* method (Silver et al., 2006). Since different programs employ different algorithms, they assigned different rankings to the same candidate reference genes (Supplementary Table S2). Similar observations were also reported in previous studies in which different reference genes were selected (Jia et al., 2019; Jin et al., 2019; Dudziak et al., 2020; Fu et al., 2022). To have a comprehensive view of expression stability, we employed RefFinder (Xie et al., 2012), in which an overall final ranking of the genes was generated based on the rankings in geNorm, Normfinder, BestKeeper, and the comparative Δ*C_t_* method.

The overall stability of the mRNA levels of the candidate reference genes was most closely reflected by the results from geNorm. The *M* values of the reference genes determined in previous studies using RNA-seq approaches ranged from 0.3 to 2.1 (Jin et al., 2019; Dudziak et al., 2020; Mao et al., 2021; Fu et al., 2022), compared to *M* values of 0.046–0.350 under *Pst* DC3000 infection, 0.078–0.524 with JA treatment, 0.082–0.790 with SA treatment, and 0.142–0.929 with ABA treatment in this study (Supplementary Table S2). As lower *M* values indicate higher expression stability, our results from this study showed that the candidate reference genes discovered here have a higher stability than the previously reported ones.

The results from NormFinder, BestKeeper, and the comparative Δ*C*_*t*_ method all showed that these candidate reference genes had the highest expression stability upon *Pst* DC3000 infection compared to other treatments. This is to be expected, as these candidates were discovered based on the proteomic dataset upon *Pst* DC3000 infection. Although the stabilities of the candidate reference genes from other treatments were lower than those with *Pst* DC3000 infection, all the genes in all the treatments, except *FBA8* with SA treatment, are regarded as suitable reference genes, according to the algorithm of BestKeeper.

Among the 18 candidate reference genes, only *ACT7* (Czechowski et al., 2005) in *A. thaliana* and *OVA6* in potato (Castro-Quezada et al., 2013) were previously reported to be suitable for being used as reference genes for RT-qPCR in multiple-treatment experiments. Based on the overall final ranking generated using RefFinder, *ACT7* was out-performed by *TIF3D1*, *RPS6B*, *FKBP15-1*, and *FBA8* in *Pst* DC3000 infection, by *RPS6B*, *PSBQ2*, and *FKBP15-1* in JA treatment, by all candidate reference genes except *RPS6B*, *OVA6*, *FKBP15-1*, *HSP70-9*, and *FBA8* in SA treatment, and by *PRPL17*, *PSBQ2*, and *FKBP15-1* in ABA treatment (Supplementary Table S2). In previous studies on *A. thaliana*-*Pst* DC3000 interaction, depending on the stress responses, the plant samples may be harvested from 1 to 5 dpi (Mackey et al., 2003; Chow and Ng, 2017; Zhang et al., 2017; Cheng et al., 2022). Although the current study only addressed the expression stability of the candidate reference genes between 0 dpi and 3 dpi, the general expression stability of the genes upon various treatments may suggest the potential of these candidate reference genes for expression normalization at different time points after *Pst* DC3000 infection. For *A. thaliana*, *Pst* DC3000 has been commonly employed as the model bacterial pathogen (Xin and He, 2013). Besides being used as the model for studying plant-bacterium interaction, *A. thaliana* is also commonly used as the model for other plant-pathogen interactions. For example, *A. thaliana*-*Hyaloperonospora arabidopsidis*, *A. thaliana*-*Alternaria brassicicola* conidia, and *A. thaliana*-*Cucumber mosaic virus* have been employed as the models for plant-oomycete interaction, plant-fungus interaction, and plant-virus interactions, respectively, (Coates and Beynon, 2010; Pochon et al., 2012; Montes et al., 2019). Although the expression stability of the candidate reference genes was not tested upon the infection by the other pathogens, the expression stability of these candidate reference genes upon the treatment of various stress hormones was demonstrated. Since JA, SA, and ABA are major hormones regulating plant-pathogen interactions (Ku et al., 2018), the results may suggest the potential of these candidate reference genes for expression normalization upon the infection by other pathogens.

In conclusion, the results in this study suggest that mass spectrometry-based LFQ in proteomic analysis is an effective approach for mining proteins and their corresponding mRNAs with stable expression levels under different conditions. Compared to RNA-seq, mass spectrometry-based LFQ does not involve amplification steps which have been known to create biases that affect the accuracy of quantification. In addition, genes involved in the maintenance of basic cellular functions generally have stable expression levels. These factors enable the identification of elite reference genes from the proteomic dataset under different experimental treatments. The expression stability of the candidate reference genes in various treatments may suggest the potential of the genes to be employed as the reference genes in treatment conditions yet to be covered in the current study. The homologs of the candidate reference genes in other plant species may also be the potential candidates of reference genes for expression studies.

## Data availability statement

The data presented in the study are deposited in PRIDE (PRoteomics IDEntifications Database, accession number PXD035677).

## Author contributions

S-SC, Y-SK, and M-YC conducted the experiments and analyzed the data. S-SC and Y-SK drafted the manuscript. Y-SK and H-ML finalized the manuscript. H-ML acquired the funding. All authors contributed to the article and approved the submitted version.

## Funding

This work was supported by the Hong Kong Research Grants Council: General Research Fund (14164617).

## Conflict of interest

The authors declare that the research was conducted in the absence of any commercial or financial relationships that could be construed as a potential conflict of interest.

## Publisher’s note

All claims expressed in this article are solely those of the authors and do not necessarily represent those of their affiliated organizations, or those of the publisher, the editors and the reviewers. Any product that may be evaluated in this article, or claim that may be made by its manufacturer, is not guaranteed or endorsed by the publisher.
