# Supplementary material for: Identification of stably expressed reference genes for expression studies in Arabidopsis thaliana using mass spectrometry-based label-free quantification
Source: Front Plant Sci. 2022 Sep 29;13:1001920. doi: 10.3389/fpls.2022.1001920 (PMC9557097; doi:10.3389/fpls.2022.1001920)
Supplement: Supplementary file 1 [file Data_Sheet_1.docx]

Supplementary Material

# Supplementary Table

**Supplementary Table 1. The IDs of the candidate reference genes and the primer sets for RT-qPCR used in this study.**

|  | Gene | Gene ID | Forward primer (5’-3’) | Reverse primer (5’-3’) | References |
| --- | --- | --- | --- | --- | --- |
| Candidate reference genes reported in this study | *ACT7* | AT5G09810 | AGCACCTTCCAACAGATGTGGA | ATAAGACAAGACACACTTAGAAGCA |  |
|  | *FKBP18* | AT1G20810 | AACTGCTCAGGTGCATTTTGA | TTGAACTCGTAAGGCTGAGCA |  |
|  | *CYFBP* | AT1G43670 | TACGTAGGAAGTATGGTAGCTGA | AACTTCATACAAGACACGCAAT |  |
|  | *FBA6* | AT2G36460 | TCTTGGTCAGATGCAAAGCT | AGCCTCTTTCTTCAGTACTTGT |  |
|  | *FKBP15-1* | AT3G25220 | ATTCTAACATTAGCTTATGCGAAGA | TCAGTTAACTTTCCCCGATAGTGA |  |
|  | *G6PDH5* | AT3G27300 | TTCTTGGTGCTTCTGGTGAT | ATCAACAAGATATCCGCGGA |  |
|  | *RPL12A* | AT3G27830 | ACTAGCTTGGCGTTGAAGGA | ACTGCAATAAGCACTTAAGCAAT |  |
|  | *RPL34C* | AT3G28900 | CAAAACTCCTGGTGGAAAATTGA | TAACAGCAACTCCAGATAGAACA |  |
|  | *FBA8* | AT3G52930 | AGAGTTCCGATGGTAAGCTT | AGCTTCGTAGTACTTCTTGCA |  |
|  | *PRPL17* | AT3G54210 | AGAAACAGATTGTTCACGCTCT | AGAGATATTGACCTTAGACAAGCT |  |
|  | *PSBQ2* | AT4G05180 | ACCATTGACAACTTGGACTAT | AGTCTTTCTTCATTAACCGAGCT |  |
|  | *TIF3D1* | AT4G20980 | TGATTACGTTGAGGAACCGT | TTATAACCATCAAGCTTGAGCAT |  |
|  | *HSP70-9* | AT4G37910 | TAATCCAATCAAATGGCCTCCGTC | AGGCCTCGAGCAGAAAGG |  |
|  | *RPS6B* | AT5G10360 | ATCAAAAACTCCGCGCCTTTTT | TGGAGTGAGAACTCCTTGTTTCAT |  |
|  | *PRPL19* | AT5G47190 | TTGTGCAAACATGGCGACGA | AAACTGATAGTCTTGAGTTCACT |  |
|  | *OVA6* | AT5G52520 | AGAAACTTGTGGTTCGGCCTA | ACATTGGCCCACTGATTAAT |  |
|  | *EIF(ISO)4G1* | AT5G57870 | AGCTTAAAGAGACCACTCAACT | TTGAACGGGAACGACACTTTCT |  |
|  | *BGAL6* | AT5G63800 | ATTCTTTCTCAGATCGAGAACGA | AGGTATTGATCACGGGATCAGGA |  |
| Reference genes commonly used in previous studies | ACT1 | AT2G37620 | CATCAGGAAGGACTTGTACGG (ACT1_1) | GATGGACCTGACTCGTCATAC (ACT1_1) | (Kim and Hwang, 2014) |
|  |  |  | GGCGATGAAGCTCAATCCAAACG (ACT1_2) | GGTCACGACCAGCAAGATCAAGACG (ACT1_2) | (Zhang et al., 2015) |
|  | ACT2 | AT3G18780 | AGGTCCAGGAATCGTTCACAGA | CCCCAGCTTTTTAAGCCTTTGA | (Chen et al., 2014) |
|  | ACT8 | AT1G49240 | CCCAAAAGCCAACAGAGAGA | CATCACCAGAGTCCAACACAAT | (Cheong et al., 2014; Jia et al., 2018) |
|  | EF-1 | AT1G07920 | TGAGCACGCTCTTCTTGCTTTCA | GGTGGTGGCATCCATCTTGTTACA | (Huang et al., 2013) |
|  | EF1α | AT1G07930 | GACATGAGGCAGACTGTTGCA | CCGGTTGGGTCCTTCTTGT | (Jeong et al., 2011) |
|  | PP2AA3 | AT1G13320 | GGTTACAAGACAAGGTTCACTC | CATTCAGGACCAAACTCTTCAG | (Huot et al., 2017) |
|  | TIP41 | AT4G34270 | TGAACTGGCTGACAATGGAGTG | CATGAGCTTGGCATGACTCTCAC | (Czechowski et al., 2005; Trdá et al., 2019) |
|  | TUB2 | AT5G62690 | CTCTCAAACTCACTACCCCCAG (TUB2_1) | AGAGGAGCAAAACCCACCAT (TUB2_1) | (Wu et al., 2019) |
|  |  |  | GAGCCTTACAACGCTACTCTGTCTGT (TUB2_2) | ACACCAGACATAGTAGCAGAAATCA (TUB2_2) | (Gao et al., 2022) |
|  | UBC21 | AT5G25760 | CTGCGACTCAGGGAATCTTCTAA (UBC21_1) | TTGTGCCATTGAATTGAACCC (UBC21_1) | (Cuéllar Pérez et al., 2014) |
|  |  |  | TCAAATGGACCGCTCTTATC (UBC21_2) | CACAGACTGAAGCGTCCAAG (UBC21_2) | (Gao et al., 2022) |
|  | UBQ5 | AT3G62250 | GTGGTGCTAAGAAGAGGAAGA | TCAAGCTTCAACTCCTTCTTT | (Cheong et al., 2014) |
|  | UBC9 | AT4G27960 | TCCTACTTCATGTAGCGCAGGAC | TCCTCCAGAATAAGGGCTATCCG | (Cui et al., 2021; Romero-Pérez et al., 2021) |
|  | UBQ10 | AT4G05320 | GGCCTTGTATAATCCCTGATGAAT | AAAGAGATAACAGGAACGGAAACA | (Huang et al., 2013) |

**Table S2. Ranking of expression stability for the 18 candidate reference genes.** The expression stability values of the 18 candidate reference genes were tested under **(A)** *Pst* DC3000 treatment, **(B)** JA treatment, **(C)** SA treatment, and **(D)** ABA treatment, using geNorm, NormFinder, Bestkeeper, comparative ΔC_t_ method, and RefFinder, and ranked accordingly.

| **(A) Rankings of expression stability for the candidate reference genes under *Pst* DC3000 treatment.** | | | | | | | | | | | |
| --- | --- | --- | --- | --- | --- | --- | --- | --- | --- | --- | --- |
| **Gene** | **geNorm** | | **NormFinder** | | **BestKeeper** | | | **Comparative ΔC_t_ method** | | **RefFinder** | |
|  | Average stability value (*M*) | Rank | Stability value | Rank | Standard deviation | Coefficient of variation | Rank | Average  standard deviation | Rank | Geometric mean | Rank |
| *ACT7* | 0.112 | 5 | 0.103 | 4 | 0.09 | 0.357 | 5 | 0.27 | 4 | 4.47 | 5 |
| *BGAL6* | 0.167 | 7 | 0.221 | 11 | 0.168 | 0.572 | 8 | 0.33 | 11 | 9.07 | 10 |
| *CYFBP* | 0.210 | 11 | 0.159 | 8 | 0.157 | 0.597 | 7 | 0.3 | 6 | 7.8 | 8 |
| *FBA6* | 0.147 | 6 | 0.213 | 10 | 0.132 | 0.506 | 6 | 0.32 | 10 | 7.75 | 7 |
| *FBA8* | 0.072 | 4 | 0.112 | 5 | 0.065 | 0.259 | 2 | 0.28 | 5 | 3.76 | 4 |
| *G6PDH5* | 0.254 | 14 | 0.331 | 14 | 0.207 | 0.72 | 9 | 0.39 | 14 | 12.54 | 12 |
| *HSP70-9* | 0.302 | 16 | 0.448 | 16 | 0.25 | 0.881 | 13 | 0.47 | 16 | 15.19 | 15 |
| *PSBQ2* | 0.225 | 12 | 0.259 | 12 | 0.26 | 1.196 | 15 | 0.34 | 12 | 12.69 | 13 |
| *EIF(ISO)4G1* | 0.325 | 17 | 0.509 | 17 | 0.3 | 1.057 | 16 | 0.52 | 17 | 16.74 | 17 |
| *TIF3D1* | 0.060 | 3 | 0.041 | 1 | 0.062 | 0.225 | 1 | 0.26 | 1 | 1.32 | 1 |
| *RPS6B* | 0.046 | 1 | 0.066 | 2 | 0.089 | 0.33 | 4 | 0.27 | 2 | 2 | 2 |
| *RPL12A* | 0.193 | 9 | 0.159 | 7 | 0.227 | 0.693 | 12 | 0.3 | 8 | 8.82 | 9 |
| *PRPL17* | 0.350 | 18 | 0.528 | 18 | 0.47 | 1.876 | 18 | 0.55 | 18 | 18 | 18 |
| *PRPL19* | 0.278 | 15 | 0.436 | 15 | 0.36 | 1.372 | 17 | 0.47 | 15 | 15.48 | 16 |
| *RPL34C* | 0.202 | 10 | 0.173 | 9 | 0.218 | 0.78 | 11 | 0.3 | 9 | 9.72 | 11 |
| *OVA6* | 0.180 | 8 | 0.153 | 6 | 0.21 | 0.726 | 10 | 0.3 | 7 | 7.61 | 6 |
| *FKBP15-1* | 0.046 | 1 | 0.095 | 3 | 0.085 | 0.311 | 3 | 0.27 | 3 | 2.28 | 3 |
| *FKBP18* | 0.236 | 13 | 0.283 | 13 | 0.255 | 0.877 | 14 | 0.36 | 13 | 13.24 | 14 |
| **(B) Rankings of expression stability for the candidate reference genes under JA treatment.** | | | | | | | | | | | |
| **Gene** | **geNorm** | | **NormFinder** | | **BestKeeper** | | | **Comparative ΔC_t_ method** | | **RefFinder** | |
|  | Average stability value (*M*) | Rank | Stability value | Rank | Standard deviation | Coefficient of variation | Rank | Average  standard deviation | Rank | Geometric mean | Rank |
| *ACT7* | 0.149 | 3 | 0.332 | 8 | 0.165 | 0.715 | 4 | 0.45 | 6 | 4.9 | 4 |
| *BGAL6* | 0.524 | 18 | 0.765 | 18 | 0.75 | 2.468 | 17 | 0.82 | 18 | 17.74 | 18 |
| *CYFBP* | 0.351 | 12 | 0.291 | 6 | 0.467 | 1.919 | 14 | 0.48 | 7 | 9.17 | 12 |
| *FBA6* | 0.185 | 5 | 0.502 | 16 | 0.168 | 0.722 | 5 | 0.57 | 14 | 8.65 | 11 |
| *FBA8* | 0.428 | 15 | 0.486 | 15 | 0.421 | 1.697 | 13 | 0.62 | 16 | 14.71 | 16 |
| *G6PDH5* | 0.487 | 17 | 0.699 | 17 | 0.77 | 2.559 | 18 | 0.76 | 17 | 17.24 | 17 |
| *HSP70-9* | 0.403 | 14 | 0.389 | 9 | 0.56 | 1.949 | 16 | 0.53 | 12 | 12.47 | 14 |
| *PSBQ2* | 0.203 | 6 | 0.203 | 5 | 0.072 | 0.331 | 1 | 0.41 | 4 | 3.31 | 2 |
| *EIF(ISO)4G1* | 0.378 | 13 | 0.319 | 7 | 0.385 | 1.346 | 12 | 0.5 | 8 | 9.67 | 13 |
| *TIF3D1* | 0.214 | 7 | 0.467 | 14 | 0.15 | 0.557 | 3 | 0.55 | 13 | 7.86 | 10 |
| *RPS6B* | 0.238 | 8 | 0.088 | 1 | 0.185 | 0.701 | 6 | 0.39 | 1 | 2.63 | 1 |
| *RPL12A* | 0.078 | 1 | 0.412 | 10 | 0.21 | 0.652 | 8 | 0.51 | 10 | 5.32 | 6 |
| *PRPL17* | 0.174 | 4 | 0.418 | 11 | 0.14 | 0.596 | 2 | 0.5 | 9 | 5.3 | 5 |
| *PRPL19* | 0.449 | 16 | 0.453 | 13 | 0.508 | 1.952 | 15 | 0.58 | 15 | 14.71 | 15 |
| *RPL34C* | 0.078 | 1 | 0.443 | 12 | 0.202 | 0.769 | 7 | 0.52 | 11 | 5.51 | 7 |
| *OVA6* | 0.324 | 11 | 0.159 | 4 | 0.32 | 1.091 | 10 | 0.43 | 5 | 6.85 | 9 |
| *FKBP15-1* | 0.263 | 9 | 0.112 | 2 | 0.26 | 0.924 | 9 | 0.4 | 2 | 4.24 | 3 |
| *FKBP18* | 0.295 | 10 | 0.121 | 3 | 0.357 | 1.251 | 11 | 0.41 | 3 | 5.61 | 8 |
| **(C) Rankings of expression stability for the candidate reference genes under SA treatment.** | | | | | | | | | | | |
| **Gene** | **geNorm** | | **NormFinder** | | **BestKeeper** | | | **Comparative ΔC_t_ method** | | **RefFinder** | |
|  | Average stability value (*M*) | Rank | Stability value | Rank | Standard deviation | Coefficient of variation | Rank | Average  standard deviation | Rank | Geometric mean | Rank |
| *ACT7* | 0.607 | 14 | 0.597 | 10 | 0.502 | 2.188 | 8 | 0.79 | 13 | 10.98 | 13 |
| *BGAL6* | 0.217 | 3 | 0.653 | 13 | 0.521 | 1.667 | 10 | 0.77 | 11 | 8.09 | 10 |
| *CYFBP* | 0.456 | 11 | 0.266 | 1 | 0.36 | 1.578 | 2 | 0.66 | 3 | 2.85 | 2 |
| *FBA6* | 0.331 | 8 | 0.296 | 3 | 0.505 | 2.129 | 9 | 0.65 | 2 | 4.56 | 3 |
| *FBA8* | 0.790 | 18 | 1.333 | 18 | 1.125 | 4.661 | 18 | 1.39 | 18 | 18 | 18 |
| *G6PDH5* | 0.357 | 9 | 0.595 | 9 | 0.433 | 1.465 | 7 | 0.78 | 12 | 9.08 | 12 |
| *HSP70-9* | 0.716 | 17 | 0.795 | 16 | 0.712 | 2.458 | 17 | 0.91 | 17 | 16.74 | 17 |
| *PSBQ2* | 0.311 | 7 | 0.592 | 8 | 0.58 | 2.517 | 11 | 0.76 | 10 | 8.86 | 11 |
| *EIF(ISO)4G1* | 0.262 | 4 | 0.469 | 7 | 0.385 | 1.291 | 4 | 0.69 | 5 | 4.86 | 5 |
| *TIF3D1* | 0.284 | 5 | 0.294 | 2 | 0.361 | 1.29 | 3 | 0.62 | 1 | 2.34 | 1 |
| *RPS6B* | 0.654 | 15 | 0.73 | 14 | 0.602 | 2.252 | 14 | 0.86 | 14 | 14.24 | 14 |
| *RPL12A* | 0.293 | 6 | 0.428 | 6 | 0.42 | 1.357 | 6 | 0.67 | 4 | 5.42 | 6 |
| *PRPL17* | 0.559 | 13 | 0.404 | 5 | 0.357 | 1.658 | 1 | 0.7 | 7 | 4.62 | 4 |
| *PRPL19* | 0.518 | 12 | 0.383 | 4 | 0.42 | 1.626 | 5 | 0.69 | 6 | 6.16 | 9 |
| *RPL34C* | 0.082 | 1 | 0.64 | 11 | 0.598 | 2.37 | 13 | 0.75 | 8 | 5.82 | 7 |
| *OVA6* | 0.379 | 10 | 0.805 | 17 | 0.671 | 2.251 | 16 | 0.89 | 16 | 14.44 | 15 |
| *FKBP15-1* | 0.688 | 16 | 0.759 | 15 | 0.606 | 2.266 | 15 | 0.88 | 15 | 15.24 | 16 |
| *FKBP18* | 0.082 | 1 | 0.644 | 12 | 0.593 | 2.097 | 12 | 0.76 | 9 | 6 | 8 |
| **(D) Rankings of expression stability for the candidate reference genes under ABA treatment.** | | | | | | | | | | | |
| **Gene** | **geNorm** | | **NormFinder** | | **BestKeeper** | | | **Comparative ΔC_t_ method** | | **RefFinder** | |
|  | Average stability value (*M*) | Rank | Stability value | Rank | Standard deviation | Coefficient of variation | Rank | Average  standard deviation | Rank | Geometric mean | Rank |
| *ACT7* | 0.367 | 8 | 0.316 | 1 | 0.31 | 1.274 | 2 | 0.75 | 4 | 2.83 | 4 |
| *BGAL6* | 0.929 | 18 | 1.18 | 18 | 0.938 | 3.114 | 18 | 1.33 | 18 | 18 | 18 |
| *CYFBP* | 0.721 | 13 | 0.82 | 13 | 0.62 | 2.467 | 15 | 1.01 | 13 | 13.47 | 14 |
| *FBA6* | 0.344 | 7 | 0.528 | 6 | 0.389 | 1.551 | 6 | 0.81 | 6 | 6.24 | 7 |
| *FBA8* | 0.821 | 15 | 0.835 | 14 | 0.545 | 2.238 | 11 | 1.02 | 14 | 13.41 | 13 |
| *G6PDH5* | 0.852 | 16 | 0.864 | 16 | 0.665 | 2.384 | 16 | 1.05 | 15 | 15.74 | 17 |
| *HSP70-9* | 0.442 | 10 | 1.041 | 17 | 0.771 | 2.739 | 17 | 1.12 | 17 | 14.89 | 16 |
| *PSBQ2* | 0.142 | 1 | 0.407 | 3 | 0.353 | 1.544 | 5 | 0.74 | 2 | 2.34 | 2 |
| *EIF(ISO)4G1* | 0.780 | 14 | 0.816 | 12 | 0.552 | 1.938 | 13 | 0.99 | 12 | 12.72 | 12 |
| *TIF3D1* | 0.399 | 9 | 0.778 | 11 | 0.588 | 2.122 | 14 | 0.94 | 9 | 10.57 | 11 |
| *RPS6B* | 0.634 | 12 | 0.773 | 10 | 0.497 | 1.839 | 8 | 0.97 | 10 | 9.9 | 9 |
| *RPL12A* | 0.293 | 5 | 0.394 | 2 | 0.34 | 1.019 | 3 | 0.74 | 3 | 3.08 | 5 |
| *PRPL17* | 0.142 | 1 | 0.418 | 5 | 0.285 | 1.094 | 1 | 0.76 | 5 | 2.24 | 1 |
| *PRPL19* | 0.276 | 4 | 0.63 | 7 | 0.475 | 1.735 | 7 | 0.82 | 7 | 6.09 | 6 |
| *RPL34C* | 0.879 | 17 | 0.858 | 15 | 0.515 | 1.869 | 9 | 1.06 | 16 | 13.84 | 15 |
| *OVA6* | 0.320 | 6 | 0.738 | 9 | 0.548 | 1.78 | 12 | 0.89 | 8 | 8.49 | 8 |
| *FKBP15-1* | 0.239 | 3 | 0.412 | 4 | 0.351 | 1.283 | 4 | 0.73 | 1 | 2.63 | 3 |
| *FKBP18* | 0.508 | 11 | 0.719 | 8 | 0.542 | 1.784 | 10 | 0.98 | 11 | 9.92 | 10 |

# Supplementary Figures

#
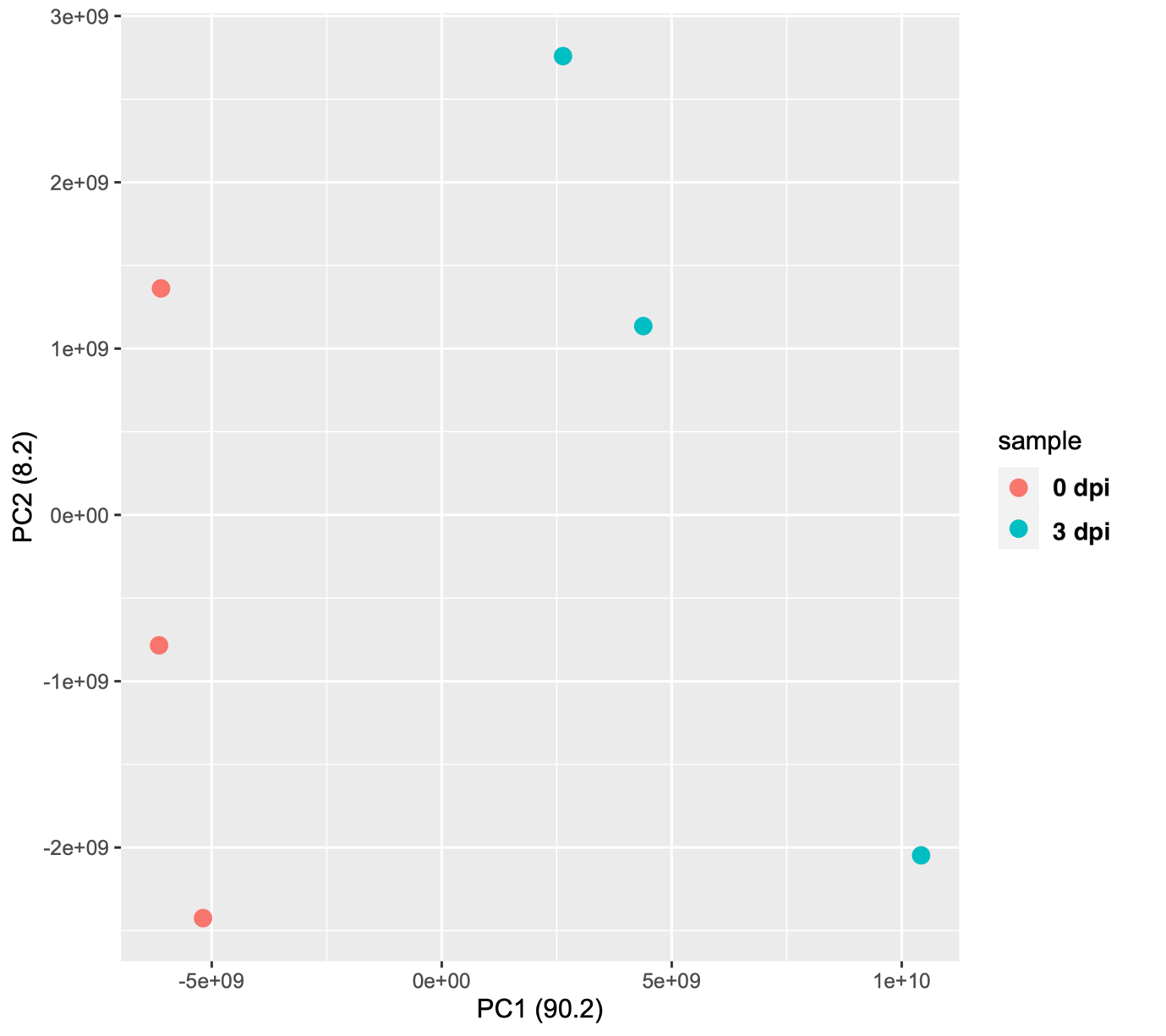


**Figure S1. Principal component analysis (PCA) of protein abundance from mass spectrometry-based label-free quantification (LFQ) calculated using Proteome Discoverer v2.4 (Thermo Fisher Scientific, Waltham, MA, USA).** The raw datafiles were search against the Arabidopsis protein database (TAIR10) with the built-in SEQUEST HT program. The PCA plot was generated using R package ggplots with default settings (Wickham, 2009). Each biological replicate was composed of three individual plants, and was represented by one dot in the PCA plot. dpi, days post-inoculation.

#
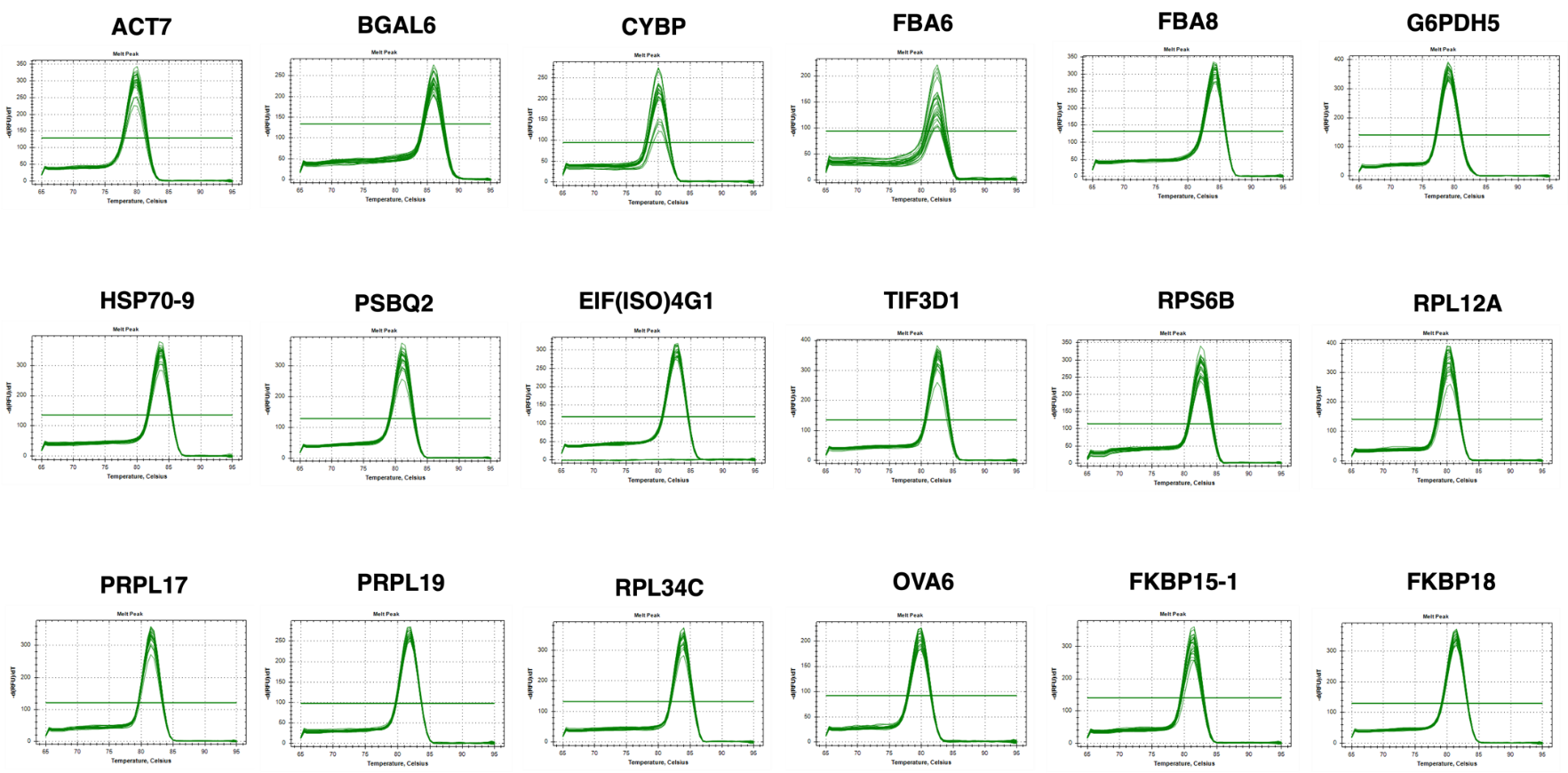


**Figure S2. Melting curve analysis for testing the amplification specificity of primer pairs.**The specificity for each primer pair was verified by melting curve analysis. Each peak map represents the melting curve of a reference gene candidate, the peaks representing the melting temperature of each gene, and the single peak indicating a single amplicon with the use of the primer pairs.


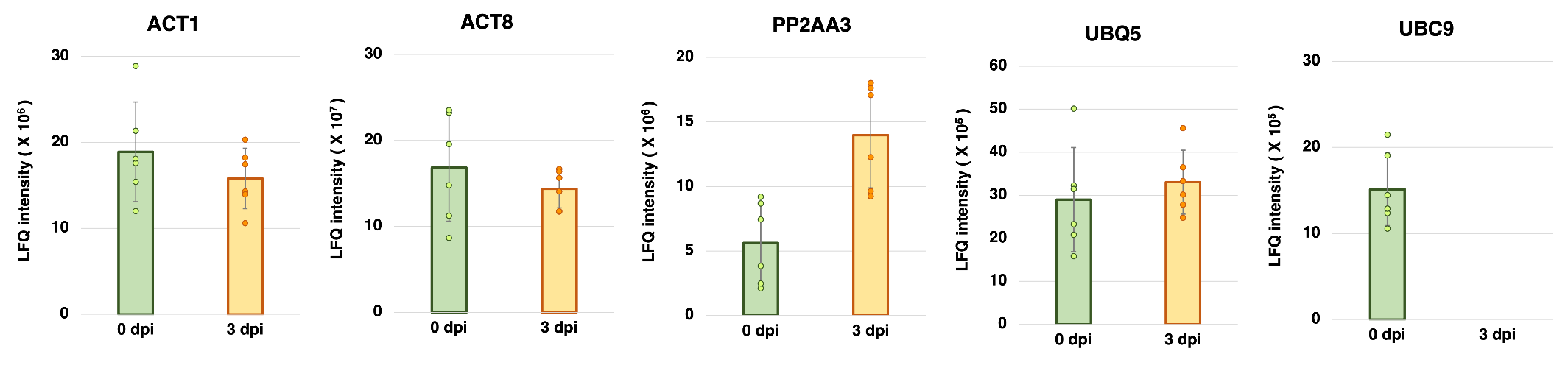


**Figure S3. Label-free quantification (LFQ) intensities of protein corresponding to reference genes commonly used in previous studies between 0 and 3 days post-inoculation (dpi) with *Pseudomonas syringae* pv. *tomato* DC3000 (*Pst* DC3000) in five-week-old *A. thaliana* ecotype Col-0.** The abundances of ACT1, ACT8, PP2AA3, and UBQ5, calculated from label-free quantification (LFQ) using Proteome Discoverer v2.4 (Thermo Fisher Scientific, Waltham, MA, USA), were not statistically different between 0 and 3 dpi (*P* > 0.05), while the abundance of UBC9 was higher at 0 dpi compared to 3 dpi (*P* < 0.05). Three individual plants were pooled as one biological replicate, each with two technical repeats of LFQ analysis. Error bar represents the standard deviation of a total of six technical repeats based on three biological replicates.


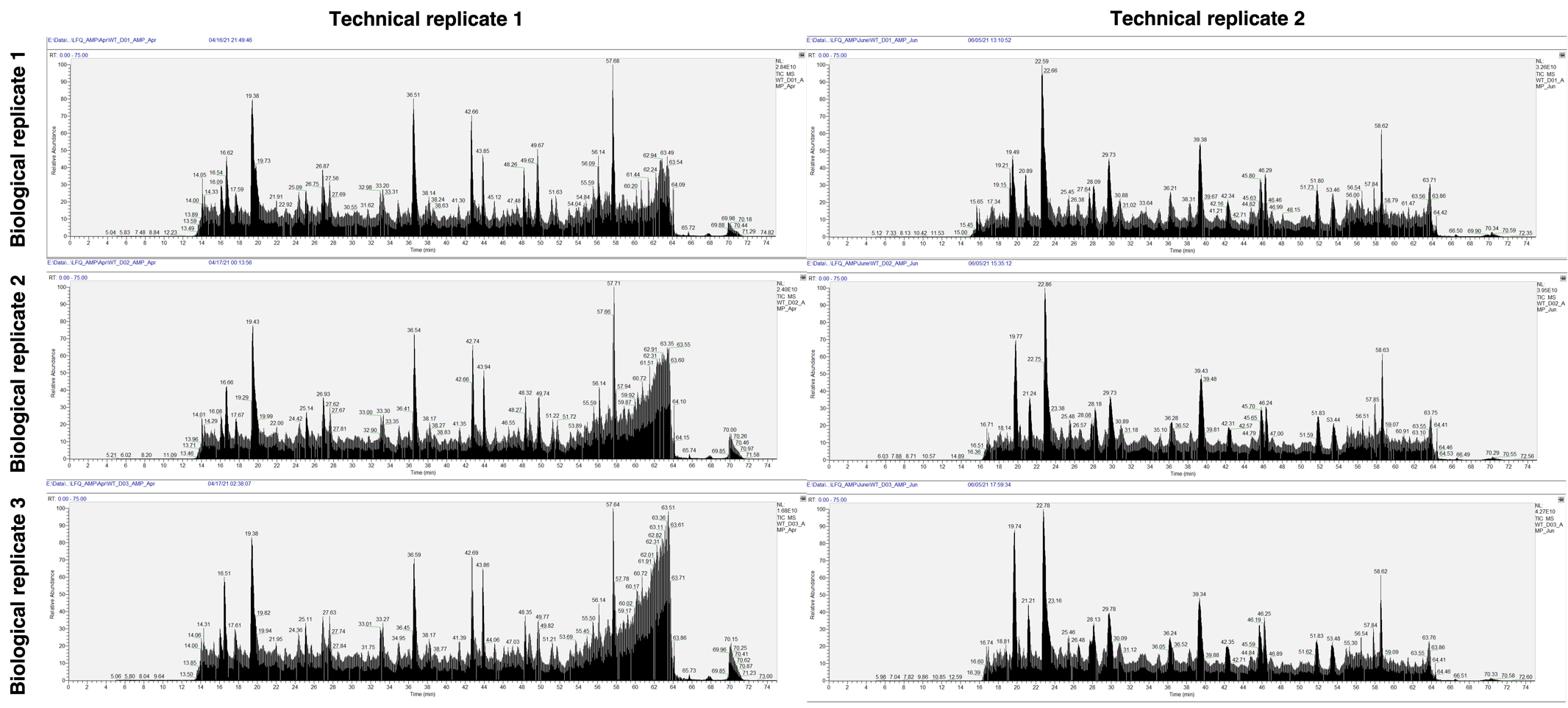


Figure S4. LC/MS-MS chromatograms of protein samples upon *Pst* DC3000 infection at 0 dpi.


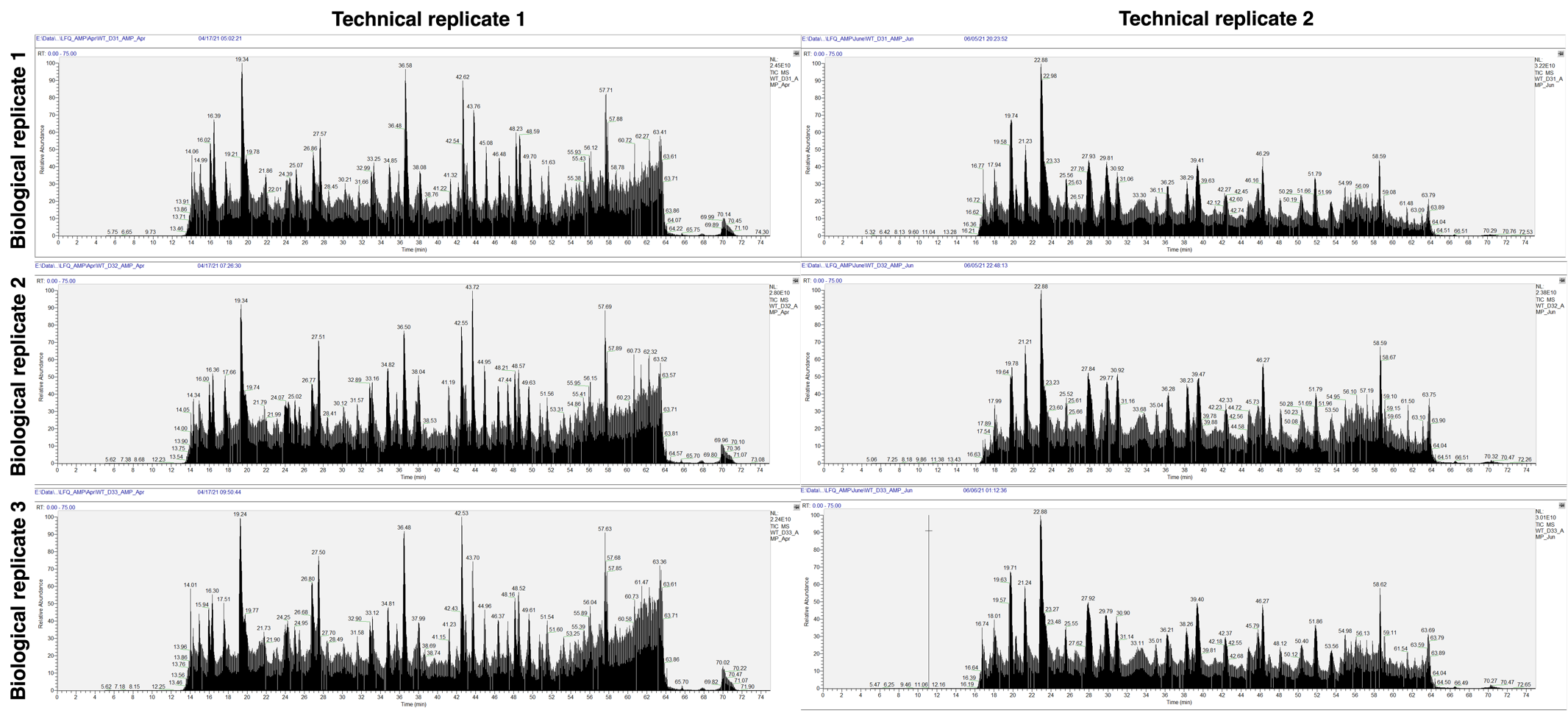


Figure S5. LC/MS-MS chromatograms of protein samples upon *Pst* DC3000 infection at 3 dpi.


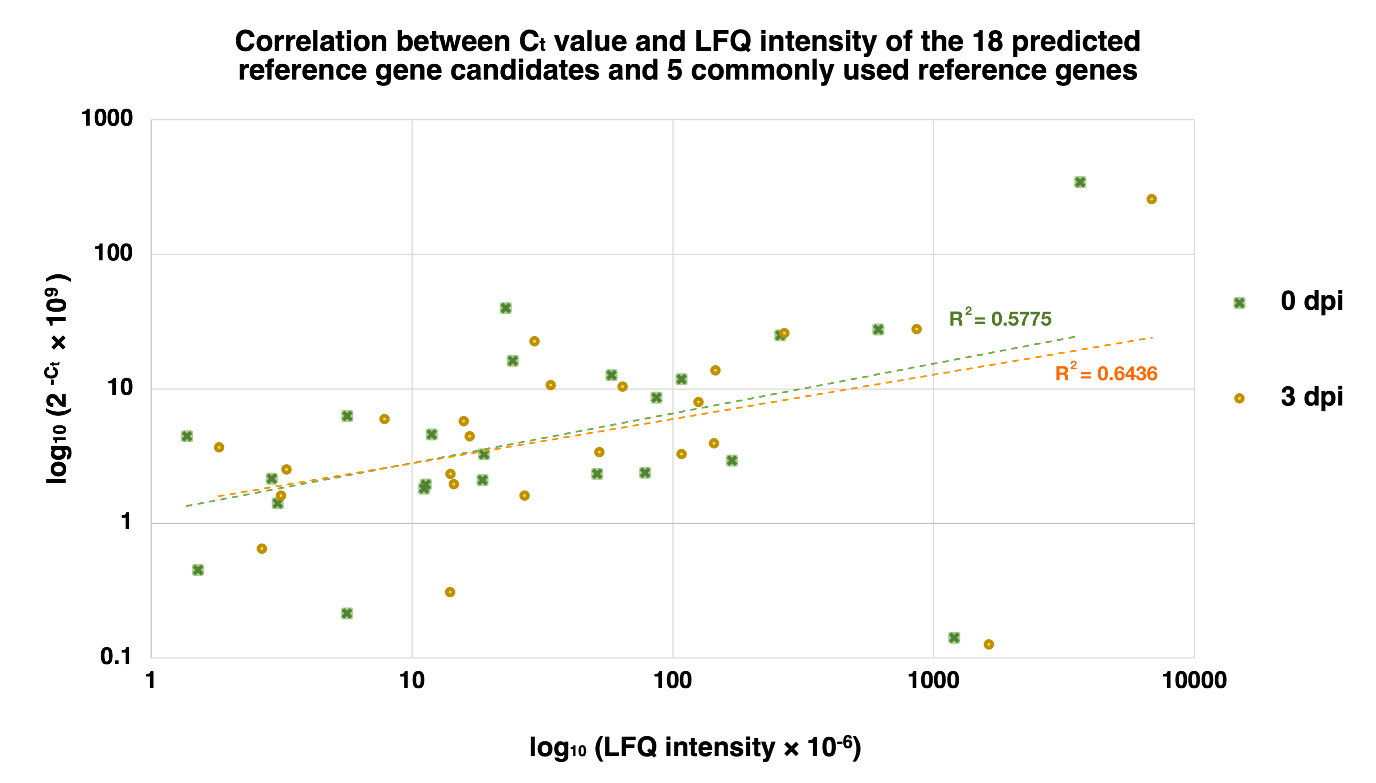


**Figure S6. The correlation between mRNA level and protein abundance.** Using the C_t_ values and the LFQ intensities of the 18 candidate reference genes predicted in this study and the five commonly used reference genes which could be quantified in the mass spectrometry-based proteomic analysis (Supplementary Figure S3), the mRNA level and the LFQ intensity was correlated. The C_t_ value used for the correlation analysis is the average value of three technical replicates from each of the two biological replicates. The LFQ intensity used for the correlation analysis is the average value of two technical replicates from each of the three biological replicates.

# Reference

Chen, C. C., Chien, W. F., Lin, N. C., and Yeh, K. C. (2014). Alternative functions of *Arabidopsis YELLOW STRIPELIKE3*: From metal translocation to pathogen defense. *PLoS One* 9, e98008. doi:10.1371/journal.pone.0098008.

Cheong, M. S., Kirik, A., Kim, J. G., Frame, K., Kirik, V., and Mudgett, M. B. (2014). AvrBsT acetylates *Arabidopsis* ACIP1, a protein that associates with microtubules and is required for immunity. *PLoS Pathog.* 10, e1003952. doi:10.1371/journal.ppat.1003952.

Cuéllar Pérez, A., Nagels Durand, A., Vanden Bossche, R., De Clercq, R., Persiau, G., Van Wees, S. C. M., et al. (2014). The non-JAZ TIFY protein TIFY8 from *Arabidopsis thaliana* is a transcriptional repressor. *PLoS One* 9. doi:10.1371/journal.pone.0084891.

Cui, B., Xu, S., Li, Y., Umbreen, S., Frederickson, D., Yuan, B., et al. (2021). The *Arabidopsis* zinc finger proteins SRG2 and SRG3 are positive regulators of plant immunity and are differentially regulated by nitric oxide. *New Phytol.* 230, 259–274. doi:10.1111/nph.16993.

Czechowski, T., Stitt, M., Altmann, T., Udvardi, M. K., and Scheible, W.-R. (2005). Genome-wide identification and teseting of superior refernece genes for transcript normalization in Arabidopsis. *Plant Physiol.* 139, 5–17. doi:10.1104/pp.105.063743.

Gao, Y., Li, Z., Yang, C., Li, G., Zeng, H., Li, Z., et al. (2022). *Pseudomonas syringae* activates *ZAT18* to inhibit salicylic acid accumulation by repressing *EDS1* transcription for bacterial infection. *New Phytol.* 233, 1274–1288. doi:10.1111/nph.17870.

Huang, T. Y., Desclos-Theveniau, M., Chien, C. T., and Zimmerli, L. (2013). *Arabidopsis thaliana* transgenics overexpressing *IBR3* show enhanced susceptibility to the bacterium *Pseudomonas syringae*. *Plant Biol. J.* 15, 832–840. doi:10.1111/j.1438-8677.2012.00685.x.

Huot, B., Castroverde, C. D. M., Velásquez, A. C., Hubbard, E., Pulman, J. A., Yao, J., et al. (2017). Dual impact of elevated temperature on plant defence and bacterial virulence in *Arabidopsis*. *Nat. Commun.* 8, 1–11. doi:10.1038/s41467-017-01674-2.

Jeong, H. J., Kim, Y. J., Kim, S. H., Kim, Y. H., Lee, I. J., Kim, Y. K., et al. (2011). Nonsense-mediated mRNA decay factors, UPF1 and UPF3, contribute to plant defense. *Plant Cell Physiol.* 52, 2147–2156. doi:10.1093/pcp/pcr144.

Jia, X., Zeng, H., Wang, W., Zhang, F., and Yin, H. (2018). Chitosan oligosaccharide induces resistance to *Pseudomonas syringae* pv. *tomato* DC3000 in *Arabidopsis thaliana* by activating both salicylic acid– and jasmonic acid–mediated pathways. *Mol. Plant Microbe Interact.* 31, 1271–1279. doi:10.1094/MPMI-03-18-0071-R.

Kim, D. S., and Hwang, B. K. (2014). An important role of the pepper phenylalanine ammonia-lyase gene (*PAL1*) in salicylic acid-dependent signalling of the defence response to microbial pathogens. *J. Exp. Bot.* 65, 2295–2306. doi:10.1093/jxb/eru109.

Romero-Pérez, A., Ameye, M., Audenaert, K., and Van Damme, E. J. M. (2021). Overexpression of F-Box Nictaba promotes defense and anthocyanin accumulation in *Arabidopsis thaliana* after *Pseudomonas syringae* infection. *Front. Plant Sci.* 12, 692606. doi:10.3389/fpls.2021.692606.

Trdá, L., Janda, M., Macková, D., Pospíchalová, R., Dobrev, P. I., Burketová, L., et al. (2019). Dual Mode of the saponin aescin in plant protection: Antifungal agent and plant defense elicitor. *Front. Plant Sci.* 10, 1448. doi:10.3389/fpls.2019.01448.

Wickham, H. (2009). “ggplot2: Elegant Graphics for Data Analysis,” in, ed. H. Wickham (New York, NY: Springer New York), 157–175. doi:10.1007/978-0-387-98141-3_9.

Wu, Z., Han, S., Zhou, H., Tuang, Z. K., Wang, Y., Jin, Y., et al. (2019). Cold stress activates disease resistance in *Arabidopsis thaliana* through a salicylic acid dependent pathway. *Plant Cell Environ.* 42, 2645–2663. doi:10.1111/pce.13579.

Zhang, H., Huang, L., Dai, Y., Liu, S., Hong, Y., Tian, L., et al. (2015). *Arabidopsis* AtERF15 positively regulates immunity against *Pseudomonas syringae* pv. *tomato* DC3000 and *Botrytis cinerea*. *Front. Plant Sci.* 6, 686. doi:10.3389/fpls.2015.00686.
